# Cortical long-range interactions embed statistical knowledge of natural sensory input: a voltage-sensitive dye imaging study

**DOI:** 10.12688/f1000research.2-51.v2

**Published:** 2013-08-02

**Authors:** Selim Onat, Dirk Jancke, Peter König

**Affiliations:** 1 Institute of Cognitive Science, Department of Neurobiopsychology, University of Osnabrück, Osnabrück, 49069, Germany; 2 Optical Imaging Group, Institut für Neuroinformatik, Ruhr-University Bochum, Bochum, Germany; 3 Cognitive Neurobiology, Bernstein Group for Computational Neuroscience, Ruhr-University Bochum, Bochum, Germany

## Abstract

How is contextual processing as demonstrated with simplified stimuli, cortically enacted in response to ecologically relevant complex and dynamic stimuli? Using voltage-sensitive dye imaging, we captured mesoscopic population dynamics across several square millimeters of cat primary visual cortex. By presenting natural movies locally through either one or two adjacent apertures, we show that simultaneous presentation leads to mutual facilitation of activity. These synergistic effects were most effective when both movie patches originated from the same natural movie, thus forming a coherent stimulus in which the inherent spatio-temporal structure of natural movies were preserved in accord with Gestalt principles of perceptual organization. These results suggest that natural sensory input triggers cooperative mechanisms that are imprinted into the cortical functional architecture as early as in primary visual cortex.

## Introduction

The early visual cortex comprises an extended and densely interwoven network, acting on millisecond time scales ^1^. Radially, activity is rapidly distributed by local feedback loops ^2, 3^. Tangentially, long, horizontal fibers enable neurons to sense regions beyond their receptive field borders ^4– 7^. Investigating the dynamics of this circuitry with simple parametric stimuli reveals well-defined selective response properties. In carnivores and primates, where these neurons are further organized in overlaid maps ^8– 11^, it is possible to satisfactorily predict changes in the layout of these maps in accord with the distribution of the stimulus energy across different visual features ^12^, but see ^13^.

However, these stimulus-response relationships can flexibly be modified by the specific connectivity patterns between distant neurons ^4, 14, 15^. For example, a surrounding stimulus which is in itself not sufficient to drive cortical neurons above firing thresholds may exert strong contextual influences on local processing ^16– 19^. These integrative phenomena are conceived as the functional backbone of various Gestalt criteria of perceptual organization ^20^ and naturally occurring visual tasks such as contour forming, figure-ground separation, object segmentation, or perceptual completion. Hence, local-to-local interactions are intrinsically tied to the integrative functionality of cortical operation. They can be conceptualized as biases originating from the cortical architecture that foster optimal coordination of large numbers of neurons in accord with the statistics of incoming signals ^21– 23^.

Today, a large body of evidence indicates that the functional properties of neurons are specifically adapted to process signals that are of ecological relevance ^24– 26^. Neuronal stimulus-response properties exhibit higher sensitivity ^27^, selectivity ^28^ and reliability ^29, 30^ in response to visual features when these are presented within their natural sensory context. However, direct functional evidence showing that cortical connectivity mediating local-to-local coupling embeds empirical statistical knowledge of natural inputs is at best scarce and it is not clear whether these interactions studied with simple stimulus configurations extrapolate to complex dynamic conditions that mimic natural input.

We recorded cortical activity using voltage-sensitive dye imaging ^31^ in response to locally presented natural movies recorded by cats in a natural habitat ^26^. We characterized contextual effects by manipulating the spatiotemporal statistical regularities between two movie-patches; we tested the hypothesis that cortical circuits responsible of contextual effects are functionally adapted to natural input statistics. Our results show that, under dynamic natural stimulation conditions, facilitatory interactions across distances beyond the classical receptive field characterize contextual effects, demonstrating that cortical circuits embed functional knowledge about the spatiotemporal relationships inherent in natural scenes.

## Materials and methods

### Stimulus conditions

**Figure 1.**
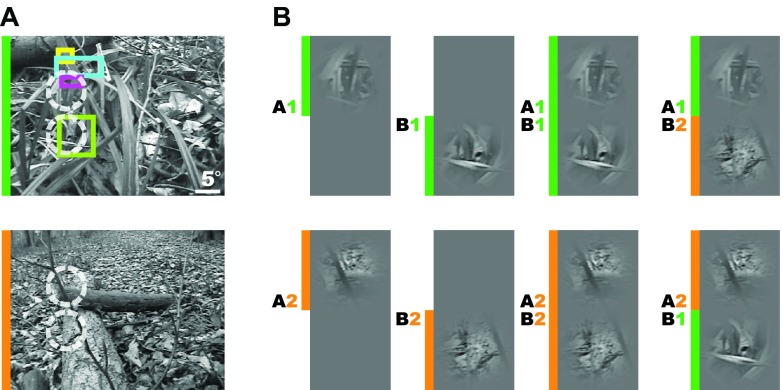
Acquisition and presentation of natural movies. ( **A**) Movie frames extracted from two different movies (marked *orange/green*) are shown. Prior to optical recordings retinotopy across the imaged cortical region was evaluated by hand mapping of receptive field locations (colored rectangles) at various penetration sites. This ensured that the distance between the mid-point of apertures was bigger than the range of classical receptive fields. ( **B**) For each experiment, two base natural movies (marked *orange/green*) were used to create different stimulation conditions. Movies were presented through either one or two Gaussian masks (3° or 4° FWHM, see Materials and Methods) centered at positions **A** (top position) or **B** (bottom position), illustrated by white circles. According to the position label ( **A** or **B**) and the index of the natural movies Movie 1 or Movie 2, the following conditions were displayed: single ( **A**1, **B**1, **A**2, **B**2), in which only one local patch was shown; coherent ( **A**1 **B**1, **A**2 **B**2), in which two local patches belonged to the same full-field movie; and incoherent ( **A**1 **B**2 and **A**2 **B**1), with local patches derived from different movies (see rightmost column).

Stimulus acquisition and presentation hardware was the same as in a previous study ^26^. Natural movies (see Figure 1A, for two example frames) were recorded at a sampling rate of 25 Hz by freely moving cats exploring a natural habitat. The recorded natural movies were presented locally, through a single or a pair of Gaussian apertures (referred to as patches in the text) for a duration of 2 s including the 200 ms prestimulus interval. We included in the analysis presented here only the initial 750 ms. Local patches were created by modulating the contrast of the movies as a function of space according to a two dimensional Gaussian function with full width at half maximum (FWHM) (~3–4°). The FWHM depended on the distance of the center point to the area centralis in individual experiments (~3–6°). The average luminance value of pixels overlapping with the two dimensional Gaussian mask were first subtracted and then multiplied pixel-wise with the Gaussian mask. This effectively reduced the luminance contrast from center to periphery. Following this step the average values were set back to the background luminance level, which was kept constant across the whole experiment.

Local conditions were indexed by two parameters: position on the screen ( **A** or **B**) and movie index (1 or 2) specifying which full-field movies were to be masked. We used different movies in different experimental sessions to increase the generalizability of our results. By displaying either one or simultaneously two local movies, the conditions **A**1, **B**1, **A**1 **B**1, and **A**2, **B**2, **A**2 **B**2 were created ( Figure 1B and Supplemental Movie S1). For conditions with a pair of patches, the distance between the centers of the two Gaussian apertures was equal to 2.5 FWHM. Local movies were corrected for mean luminance so that the average of the pixels within the Gaussian apertures was always equal to the brightness of the background in which they were embedded. However, we did not equalize the contrast within each aperture, as it is not possible to do so without introducing strong artifacts, particularly in cases where the local portion of a movie frame contains regions with homogenous brightness values belonging to object surfaces.

Prior to optical recordings, the topographic mapping between the cortical surface and the visual field were scrutinized by means of several electrode penetrations, and local stimuli were positioned so that the upper movie-patch matched the receptive field position of the simultaneously recorded multiunit activity. This ensured that the distance between the centers of the Gaussian masks extended beyond the borders of classical receptive fields.

### Experimental setup

Animals were initially anesthetized with ketamine (15 mg kg ^-1^ intramuscularly (i.m.)) and xylazine (1 mg kg ^-1^ i.m.), supplemented with atropine (0.05 mg kg ^-1^ i.m.). After tracheotomy, animals were artificially respirated, continuously anaesthetized with 0.8–1.5% isoflurane in a 1:1 mixture of O _2_/N _2_O, and fed intravenously. Heart rate, intratracheal pressure, expired CO _2_, body temperature, and electroencephalograms (EEG) were monitored during the entire experiment. The skull was opened above the primary visual cortex and the dura was resected. Paralysis was induced and maintained by alcuronium dichloride (Alloferin ^®^). Eyes were covered with zero-power contact lenses for protection. External lenses were used to focus the eyes on the screen. To control for eye drift, the position of the area centralis and receptive field positions were repeatedly measured. A stainless steel chamber was mounted and the cortex was stained for 2–3 hours with voltage-sensitive dye (RH-1691), and unbound dye was subsequently washed out with artificial cerebrospinal fluid. All surgical and experimental procedures were approved by the German Animal Care and Use Committee (AZ 9.93.2.10.32.07.032) in accordance with the Deutsche Tierschutzgesetz and NIH guidelines.

### Data acquisition and preprocessing

Optical imaging was accomplished using an Imager 3001 (Optical Imaging Inc, Mountainside, NY) and a tandem lens macroscope ^32^, 85 mm/1.2 toward camera and 50 mm/1.2 toward subject, attached to a CCD camera (DalStar, Dalsa, Colorado Springs). The camera was focused ~400 µm below the cortical surface. For detection of changes in fluorescence, the cortex was illuminated with light of 630 ± 10 nm wavelength and emitted light was high-pass filtered with a cutoff of 665 nm using a dichroic filter system. Cortical images were acquired at a frame rate of 220 Hz and covered regions of approximately 10 × 5 mm ^2^ of primary visual cortex. The relevant retinotopic region of area 18 was captured (lower contralateral quadrant of the visual field), and parts of area 17 were also occasionally captured. For electrophysiological recordings, a custom-built device was used that allowed targeted penetrations at different locations without opening the sealed recording chamber.

The raw data was processed in two steps ^26^. First, in order to remove differences in illumination across different pixels, divisive normalization was performed on all the recorded raw samples of a given pixel by its mean during prestimulus period. Second, heart-beat and respiration-related artifacts were removed by subtracting the average blank signal recorded in the absence of stimulation. These differences were later normalized by the blank signal in order to gain independence from the global activity level fluctuations occurring during the course of an experiment. As our recordings were synchronized with the heart-beat cycle of the animal, this blank subtraction step effectively removes these artifacts. Moreover, this method is preferred over the cocktail blank correction because our conditions were not composed of orthogonal stimuli. These steps were applied for each trial separately and the outcome was averaged across trials. The number of trials ranged from 25 to 35 for different experiments.

### Model fitting and statistical evaluation

We computed spatial profiles by averaging the evoked data across the temporal dimension. These were fitted with a two dimensional Gaussian function of the form:

*G*( *x*, *y*) = *A* exp(( *x* – *μ _x_*) ^2^ / *σ _x_* + ( *x* – *μ _y_*) ^2^ / *σ _y_* + ( *x* – *μ _x_*)( *y* – *μ _y_*) / *ρ*)

Where *A*, *σ _x_*, *σ _y_*, *μ _x_*, *μ _y_*, and *ρ* represent the peak value, the horizontal (medio-lateral) and vertical (antero-posterior) spreads, the position of the Gaussian function (medio-lateral or antero-posterior), and the rotational parameter, respectively. We used *lsqcurvefit* function provided by Matlab (2007b, The MathWorks, Natick, MA) using a large-scale trust-region-reflective algorithm. Prior to optimization runs, initial parameters of the Gaussian function were roughly estimated using different heuristics for each experiment and condition separately. As the fitting function we used superposition of two Gaussian functions G _1_( *x*, *y*) + G _2_( *x*, *y*) centered roughly on separate activation spots. Almost always, the activity spots were clearly distant and well-isolated from each other. All parameters were estimated simultaneously. Using two Gaussians in all stimulation conditions, including single conditions, ensured that the activation at the distant locations did not influence the fit, therefore possible cross-contributions were negligible. Furthermore the center position of each single Gaussian fit was constrained around the location of the peak responses at locations **A** and **B**. This helped avoiding complications where Gaussians could overfit noise during single stimulation conditions and this was necessary to estimate the value of the indirect activation. We also constrained the value of different parameters to avoid fitting to irrelevant cortical activity; this was most useful in the single condition case, where there was only one single activity spot.

For the statistical evaluation of confidence intervals, we used the bootstrapping method with 1000 repetitions and an alpha value of 0.05. The confidence intervals are provided within square brackets following average values. We tested the significance of median activities using the Wilcoxon sign rank test; the p-values are provided within brackets.

## Results

We performed voltage-sensitive dye imaging (VSDI) in the primary visual cortex of anesthetized cats (n = 4). Multi-unit recordings complemented these measurements and provided information on receptive field properties and localization and spatial extent (see Materials and methods). In order to investigate long-range cortical interactions under ecologically relevant dynamic stimulation conditions, natural movies were presented locally by applying either one or two Gaussian masks (3–4° FWHM) to the original full-field movies ( Figure 1A). Presentation of two movies ( Movie 1 and Movie 2) viewed through apertures at two different positions (position **A** and **B**) creates a total of 8 different local stimulation conditions ( Figure 1B, please see also Video S1): *Single* conditions ( **A**1, **B**1, **A**2 and **B**2) consisted of isolated local movie-patches that provided no contextual information ( Figure 1B, *first* and *second* column). In contrast, *coherent* ( **A**1 **B**1 and **A**2 **B**2) and *incoherent* ( **A**1 **B**2 and **A**2 **B**1) conditions provided contextual information in the form of another distant movie-patch. Here, two movie-patches stemming from either the same ( *coherent*) or different ( *incoherent*) original natural movies were presented simultaneously at two locations that were larger than the typical classical receptive field sizes ( Figure 1A, colored boxes representing receptive fields). Whereas coherent conditions leave the spatiotemporal characteristics of natural movies intact, incoherent stimulation eliminates naturally occurring correlations between apertures and induces an evident dissonance (please see Video S1).

### Effect of contextual stimulation on activity amplitudes

**Figure 2.**
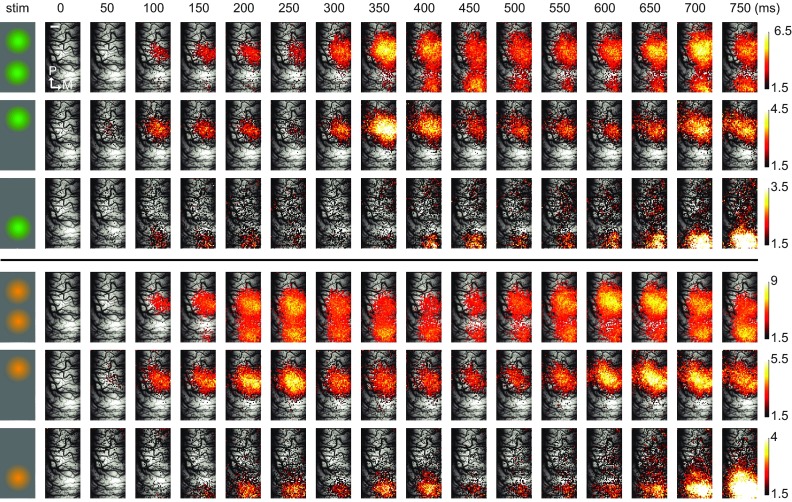
Dynamics of cortical activity evoked by locally presented natural stimuli. ( **A**) Spatiotemporal activity patterns produced by locally presented natural movies is shown overlaid on the vascular image. Leftmost column schematically shows the corresponding stimulus condition and the movie used ( *orange* and *green*). Each single frame represents the average activity across trials (n = 35) within 50 ms of non-overlapping segments of recorded data. The cortical representations of these stimuli were located along the antero-posterior axis, reflecting the vertical positioning of the movie-patches in the visual field. Different spatial extents and peak values of cortical activation were observed along the presentation duration at different frames. Please note that different color-scales are used for different conditions in order to emphasize the detailed spatial structure. Color scale represents ΔF/F (x10 ^-4^). Color scale maximum is set to 3.3 standard deviations of activity levels. Only the most active pixels corresponding to highest 25 ^th^ percentile are shown. For a video presentation please refer to the Video S2. Scale bar (shown in the first row in the frame zero) = 1 mm.

Cortical responses to two different movies presented in single and coherent conditions for a duration of 750 ms are shown as space-time plots ( Figure 2, top and bottom rows for Movie 1 and Movie 2, see also Video S2). Conditions are indicated on the left-most column. Upon localized stimulation by natural movie-patches, activity emerged from baseline level with variable delays among conditions. The cortical dynamics induced by the individual stimuli show different temporal profiles and suggest that instantaneous activity levels were determined by the specific properties of each natural movie. Each single movie evoked well-separated spots of activity on the cortical surface indicating that the thalamic input was spatially well resolved at this stimulus configuration. Furthermore, we observed large differences in the activity levels between single and coherent conditions (note the difference in color scale). In this experiment, while the peak activity (maximum activity level across all pixels) during single conditions (bottom two rows in each panel) was 6.67×10 ^-4^ ΔF/F, coherent conditions (top row in each panel) led to a value of 7.86×10 ^-4^ ΔF/F, corresponding to an increase of 18%. As the direct input to the recorded cortical region was identical during single and coherent conditions, we attribute these differences in activity to the impact of long-range interactions on cortical dynamics under natural stimulation conditions.

**Figure 3.**
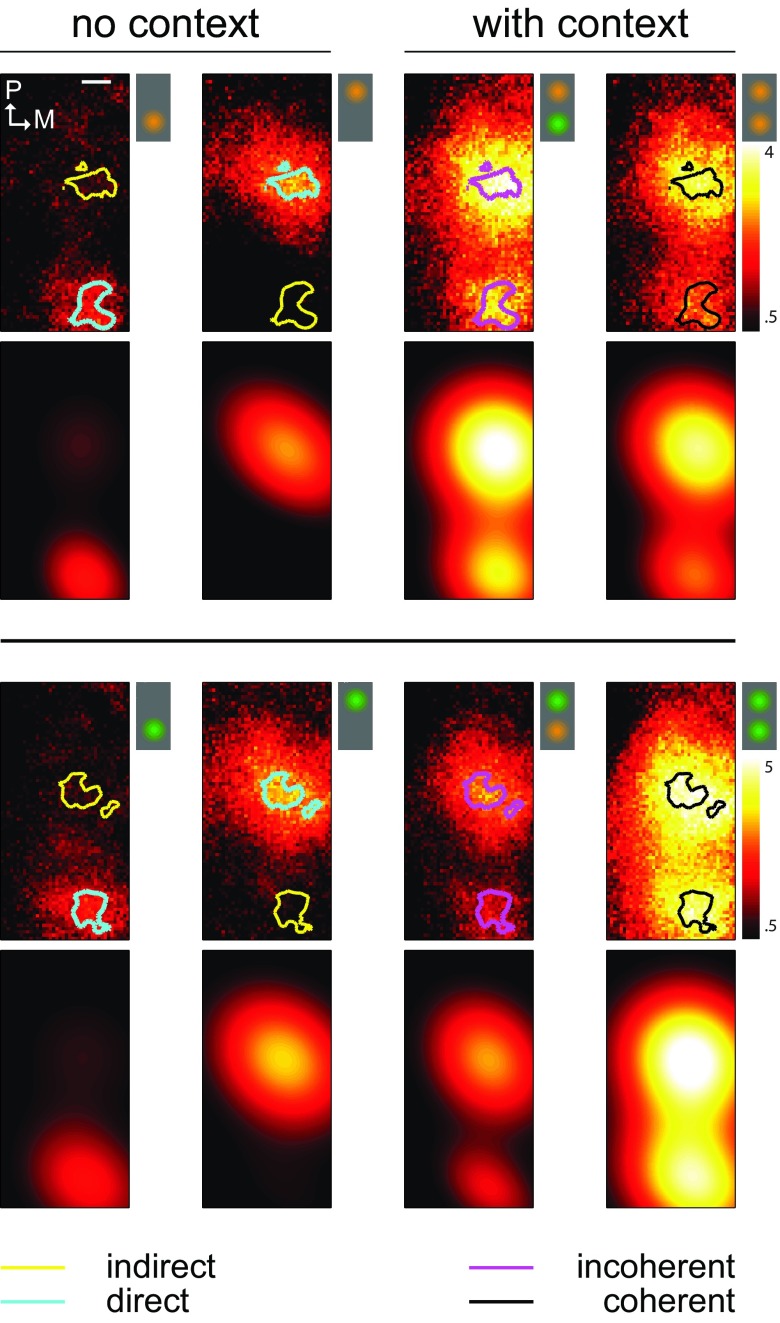
Observed and modeled spatial profiles of activity patterns. Spatial activity profiles ( *first* and *third* rows) in response to two different natural movies ( *upper* and *lower* panels) presented at various stimulation conditions with ( *third* and *fourth* columns) or without ( *first* and *second* columns) contextual information. Icons schematically represent the stimulation conditions. These are computed by time-averaging the data presented in Figure 2. Activity during no-context conditions was used to define a pair of interest regions per movie. This was done by selecting the pixels with highest activity located within the top 5 ^th^ percentile (see contour lines). Within these regions of interest we derived direct ( *cyan*), indirect ( *yellow*) and coherent ( *black*) and incoherent ( *magenta*) activity levels. The observed activity profiles were parametrically fitted using a composite function involving two 2-D Gaussians ( *second* and *fourth* rows). The goodness of fit values characterized by the correlation coefficient is shown for the whole data set in Figure 4D. Color scale represents ΔF/F (x10 ^-4^).

To quantify long-range interactions we computed spatial profiles of activity levels under stimulation conditions with or without context ( Figure 3, *first* and *third* rows). These maps represent the average activity of each single pixel across stimulation duration and demonstrate clearly the spatially restricted non-overlapping foci of activity. Note that different movies produced different amplitudes of activity. Thus, occasionally, incoherent conditions, incorporating a movie that drives the cortex more strongly, can appear more highly activated than in coherent conditions. Therefore, cross-wise comparisons (as in Figure 5B) between both incoherent conditions are needed to calculate the net interaction effects between coherent and incoherent movies.

**Figure 4.**
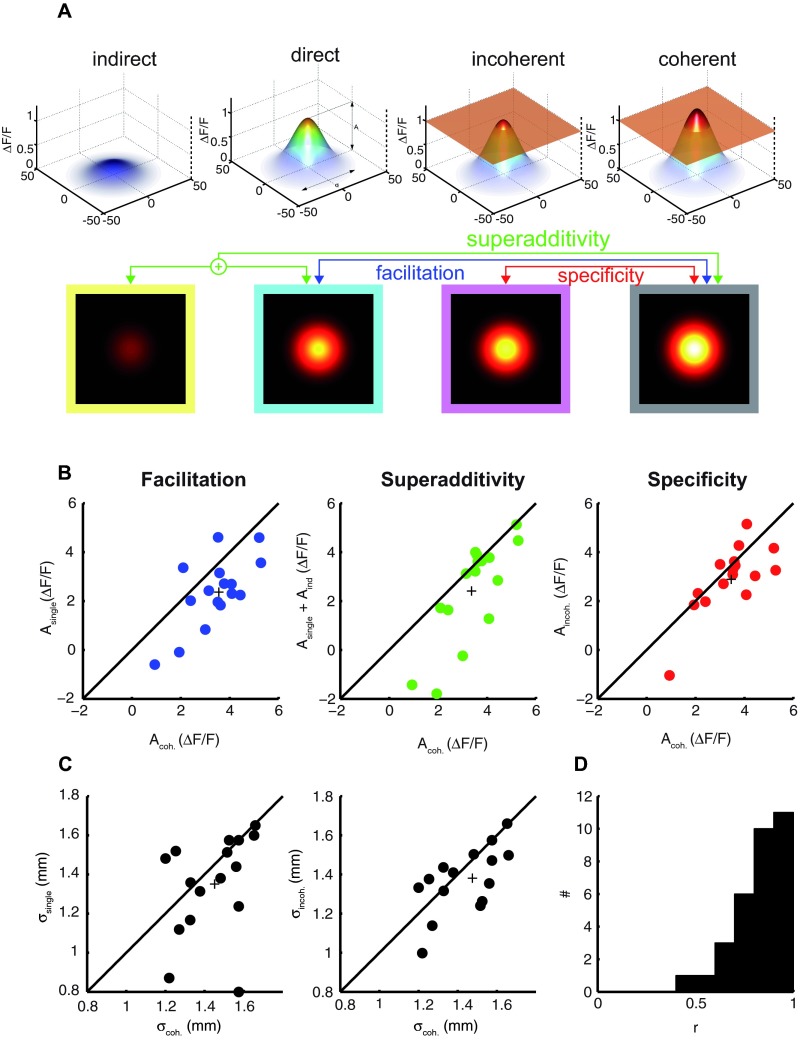
Distant natural stimulus exerts facilitatory effects on local activity levels. ( **A**) Modeled activity profiles averaged across all experiments (n = 4) and movies in response to *indirect*, *direct*, *coherent* and *incoherent* stimulation types are depicted ( *first* row, three dimensional depiction; *second* row, top view). Transparent *orange* plane ( *third* and *fourth* columns) marks the peak level of the Gaussian function representing the responses to direct stimulation at the absence of contextual information ( *second* column). We compared different parameters of the Gaussian fits across different stimulation types in order to characterize different contextual interactions (see *red*, *green*, *blue* arrows). ( **B–D**) For each of the comparisons, peak ( **B**) and joint spread values ( **C,D**) of Gaussian fits are presented as scatter plots (see matching arrow and dot colors). Each experiment contributes 4 different points. The plus sign represents the mean. ( **D**) The distribution of correlation coefficient between fits and the observed data for the whole dataset.

The precise shape of spatial activity profiles shown in Figure 3 varied considerably across different experiments. This is to be expected, as the location and extent of activity spots depend strongly on the recording conditions specific for each experimental session. It was therefore not straightforward to compare these spatial activity profiles across different experiments. We used a parametric approach in order to circumvent this problem and modeled spatial activity profiles recorded during different experiments using two-dimensional Gaussian functions with 6 free parameters. These parameters consisted of peak value ( *A*), its horizontal and vertical position ( *μ _x_* and *μ _y_*), horizontal and vertical spread ( *σ _x_* and *σ _y_*) as well as a rotational parameter ( *ρ*) (see Materials and methods). The modeled spatial activity profiles are presented together with the empirical data in Figure 3 ( *second* and *fourth* rows, same colorbar). The correlation coefficient between these fits and the empirical data was on average 0.88 [0.82, 0.91] (average, [95% bootstrap confidence intervals], same convention in the following). For the whole data set, the distribution of correlation coefficients ( Figure 4D) was negatively skewed and equal to 0.82 [0.78, 0.86] on average. Hence, compared to many thousands of pixels typically recorded in optical imaging, our parametric approach provided a major reduction in dimensionality without compromising the precise characterization of response patterns.

We computed 4 types of characteristic spatial activity profiles from 3 different stimulation conditions ( Figure 4A, *first* row, three-dimensional depiction; *second* row, top view representation). From activity during single conditions we derived the characteristic activity profiles for direct ( *cyan* border) and indirect ( *yellow* border) stimulation types. Whereas the direct activity represents the baseline responses to a single movie-patch in the absence of any contextual stimuli, the indirect activity captures the influence of an isolated distant movie-patch. Similarly we computed the characteristic activity patterns in response to movie-patches presented either in coherent ( *dark gray* border) or incoherent conditions ( *magenta* border). In Figure 4A, we visualize these four characteristic activity profiles after normalizing separately peak and spread parameters by their corresponding values obtained during direct stimulation. This was done for each experiment separately and the median fitted values were computed subsequently (this was necessary in order to eliminate outliers that originated from the normalization procedure).

We observed major changes in the characteristic activity profiles that were reflected in peak and spread parameters (compare different columns in Figure 4A). Concerning the peak activity, the indirect effect ( Figure 4A, *first* column, *yellow* borders) of a single movie-patch presented at a distant location was on average slightly excitatory; however, this was statistically not significant (sign-test = 0.8). However, it should be noted that we observed net-excitatory effects as frequently as suppressive effects with similar amplitudes at the distant non-stimulated locations. The occasional occurrence of suppression of net activity below baseline levels in the far periphery have been shown with VSDI when presenting local stimuli without contextual surround (see Figure 1 in ^33^). During the two conditions where context was present ( Figure 4A, *third* and *fourth* columns, *magenta* and *dark gray* borders), the peaks were higher than during stimulation without context (compare to *second* column, *cyan* border). Importantly, among those conditions where context was present, coherent context resulted in higher peak activity values (compare *third* and *fourth* columns).

We quantified the *total facilitation* effect by comparing the activity induced by direct and coherent stimulation ( Figure 4A, see *blue* lines). This is depicted in ( Figure 4B, *blue* dots) for each individual comparison (4 dots per experiments). In nearly all cases, the peak activity during coherent stimulation was higher than direct activity measured during single conditions. In 2 cases, a positive activation was observed only during coherent conditions. While direct drive evoked an average peak activity of 0.23×10 ^-3^ ΔF/F [0.16×10 ^-3^, 0.29×10 ^-3^ ΔF/F] (average, [95% bootstrap confidence intervals], same convention in the following) across experiments, a value of 0.34×10 ^-3^ ΔF/F [0.27×10 ^-3^, 0.39×10 ^-3^ ΔF/F] was observed during coherent input. The pairwise difference between peak values was equal to 0.11×10 ^-3^ ΔF/F [0.04×10 ^-3^, 0.14×10 ^-3^ ΔF/F], corresponding to an increase of 45.2% [19.8%, 62.3%] in peak value and this was significantly different than zero (p = 0.0011, pairwise t-test). We therefore conclude that contextual stimuli presented at distant locations have a substantial modulatory effect on local activity. We further compared the peak values between direct and incoherent stimulation conditions (not shown as a scatter plot). The presence of an incoherent context resulted in an increase of 24.5%; this increase was, however, not significantly different from zero (sign-test, p = 0.8; t-test, p = 0.08).

To what extent can the total facilitatory effect be accounted for by the indirect additive effect of the distant movie-patch? We compared the activity during coherent stimulation conditions to the predicted activity by the sum of direct and indirect responses ( Figure 4A, *green* lines). We found a superadditive effect of contextual stimulation in nearly all comparisons ( Figure 4B, *green* dots). The superadditive facilitatory effect quantified as the difference between coherent conditions and the sum of single and indirect activations corresponded to 41.9% [20.9%, 76.2%] (signtest, p = 0.004; t-test, p = 0.009), hence only about 3.3% of the contextual effect was accounted for by linear interactions. This shows that long-range interactions result to a large extent from non-linear interactions between cortical sites.

To what extent are the non-linear contextual influences adapted to the statistical regularities of natural movies? The total facilitatory effect quantified above incorporates both the specific and unspecific influences originating from contextual stimulation. While the modulation of peak activity by an incoherent context can be attributed to the unspecific effect of the distant stimuli, any incremental effect of a coherent context can be attributed to the specific adaptation of these interactions to the statistics of natural movies. To evaluate the specificity of these interactions we compared the peak values between coherent and incoherent conditions ( Figure 4A, *red* line; Figure 4B, *red* dots). We observed an increase of 20.7% [4.18%, 38.12%] in the peak activity level, and this was found to be marginally significant (sign-test, p = 0.21; t-test, p = 0.053). However, compared to the 45.2% observed for total facilitation, this analysis shows that about 54.2% of the facilitation results from specific interactions. Therefore, the non-linear facilitatory effects were fully effective only when the two movie-patches complied with the statistical regularities specific to natural movies.

### Effect of contextual stimulation on spatial extent of activity

It is possible that long-range facilitation by the contextual sources of information are accompanied by a modification of the total spatial extent of cortical activity. For example, the presence of contextual information could result in a more tuned spatial activity profile leading to a decrease in spread parameters with the presence of context. Alternatively, contextual information could potentially cause a larger number of cortical neurons to be allocated. In order to test these different hypotheses, we evaluated the influence of context on the spatial extent of activated cortical space and compared the average spread parameters ( *σ _x_* and *σ _y_*) between single and coherent conditions. The cortical activation extended larger surfaces during conditions of stimulation where context was present ( Figure 4C). We found that the presence of a coherent context increased 10.9% [1.4, 29.2%] the joint spread parameter ( Figure 4C, *left* panel). Considering each dimension separately we found that contextual information increased the spread parameter only along the direction of the context (along anterio-posterior axis). Consequently while an increase of 18.1% [5.3, 39.4%] in *σ _y_* was observed, there was no significant change in *σ _x_* [-1.9%, 24.0%]. This effect was further boosted by the presence of a coherent context. Comparing incoherent and coherent activations ( Figure 4C, *right* panel), we found a similar result. Here again only the *σ _y_* parameter was significantly different and an increase of 8.8% [0.5, 19.9%] was observed. Therefore, as in the case of peak activity modulations, coherent context had a stronger impact on the spread parameter compared to the case where the contextual information was absent or incoherent. We conclude that rather than a sharpening of the spatial profile, more cortical space is allocated when contextual information is present. Furthermore, the direction of this increase is biased towards the location of the contextual information.

### Effect of contextual stimulation on time-course of activity levels

**Figure 5.**
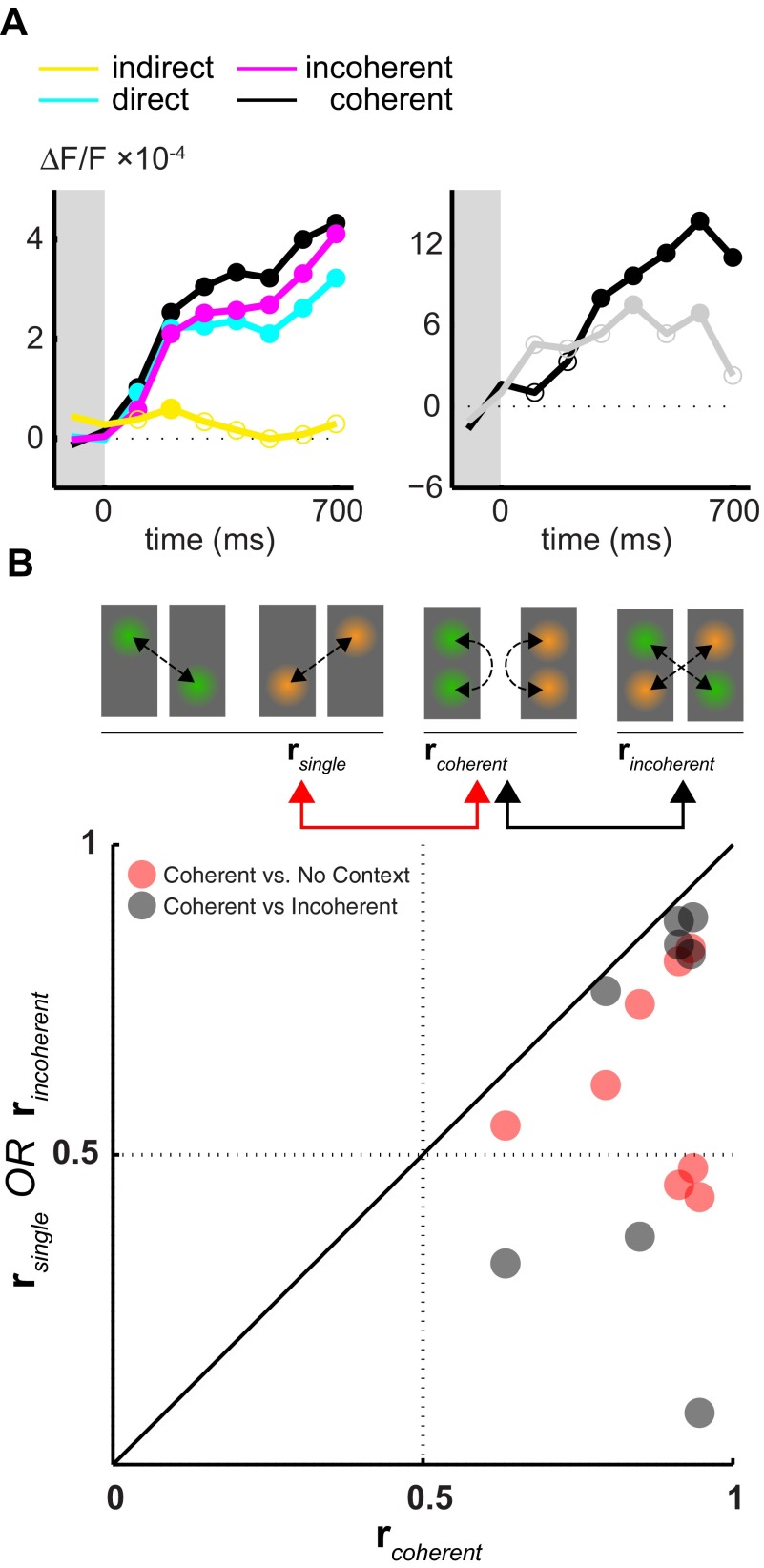
Time-course of contextual interactions. ( **A**) The temporal evolution of activity in response to indirect ( *yellow*), direct ( *cyan*), coherent ( *black*) and incoherent ( *magenta*) stimulation ( *left* panel). Average activity within the regions of interest (depicted in Figure 3) is used. For each temporal window the median across experiments and movies is presented. Median values that are statistically different (sign-rank test, alpha = 0.05) from zero are depicted with filled circles. The pair-wise difference of amplitudes between coherent and single conditions ( *black* line) as well as between coherent and incoherent ( *gray* line) conditions is shown in the right panel. ( **B**) The similarity between the time-courses of activity recorded at two distant regions of interest is evaluated for each movie separately under different conditions (2 dots per experiment). r _coherent_ measures the correlation between two time-courses of activity evoked by the simultaneous presentation of two local movie-patches. r _incoherent_ and r _single_ were computed using the activity evoked by exactly the same input however presented at different conditions (see icons and arrows). The scatter plot depicts the comparison of r _coherent_ to r _single_ and r _incoherent_.

In order to have a better grasp on the temporal unfolding of long-range interactions, we next characterized the time-course of the facilitatory effects. To this end we used the evoked activity values and limited the analysis to pixels that were most strongly driven by the movie-patches. Based on the activity profiles during two single conditions ( Figure 3, *first* and *second* rows), we defined two non-overlapping regions of interest for each movie condition by choosing those pixels that lay within the highest 5 ^th^ percentile of activity (see contour lines). These most responsive pixels were typically located centrally with respect to the activity spot. For each of the afore-mentioned activation types ( *indirect*, *direct*, *incoherent* and *coherent*) we computed the mean time-course of activity across all movies and experiments within these most strongly driven pixels ( Figure 5A, same colors as in Figure 4A and Figure 3). Please note that here experiments were conducted using different natural movies leading to the loss of a specific temporal profile. Samples of the time course of the facilitatory effect with mean activity significantly different than zero are depicted with filled circles (t-test).

As noted before, the indirect influence of the distant single movie-patch was slightly excitatory ( Figure 5A, *yellow* line). However, contrary to the previous parametric analysis, which was not temporally resolved, we detected here a significant effect of the indirect input at ~100 ms (p = 0.04, see filled circle). This confirms that the indirect influence of a movie-patch presented in isolation to its neighboring regions is of excitatory nature and occurs quickly.

During direct stimulation in the absence of context ( Figure 5A, *cyan* line), activity increased with stimulus onset and quickly reached a plateau at 100 ms, exactly where the indirect drive reached the significance level. At this point, the activity was 3.7-times stronger than the indirect drive. All samples following stimulus onset were statistically different from zero (p < 0.002). As expected, with the presence of a coherent context, the facilitatory interactions caused stronger activity levels throughout stimulus presentation. These were effective as early as 100 ms following stimulus onset ( Figure 5A, left panel, *cyan*). We computed the pair-wise differences between coherent and single conditions and evaluated whether these deviated significantly from zero level ( Figure 5A, right panel, *black* line). These facilitatory effects quickly followed after stimulus onset and reached significance around 300 ms (p < 0.049). The presence of an incoherent context had a smaller impact on activity levels (left panel, *magenta* line) and consequently the time-course of activity was similar to conditions where no context was present. The difference between coherent and incoherent conditions (right panel, *gray* line) computed over the stimulus presentation was positive throughout the stimulus presentation and reached the significance levels at two time frames (p < 0.042, see filled circles). The time-resolved analysis presented here complements the parameteric approach. We conclude that the interactions between different cortical locations occur quickly following stimulus onset and they persist across the stimulus presentation.

During single conditions, the activity within the region of interest is mainly determined by direct sub-cortical input and, therefore, the bottom-up characteristics of the input stream are presumably the sole determinants of the precise time-course. Additionally, as natural movies contain non-zero correlations across long distances, it is expected that activity profiles at locations **A** and **B** exhibit certain amount of similarity that would lead into correlations in the activity time-courses. We quantified these similarities at locations **A** and **B** during two single conditions by measuring the correlation coefficient ( Figure 5B, schematic representation). The correlations, r _single_, between the time-courses of activity recorded in both locations were never negative. Temporal resolution of the time-courses in this analysis was 200 Hz in order to capture its detailed structure. We observed an average correlation of r _single_ = 0.57 [0.47, 0.67], suggesting that low-level characteristics of movies were to a large extent common to both locations. How do the lateral interactions, which are effective during simultaneous presentation of two movie-patches, influence the precise time-course of activity? To answer this question, we computed r _coherent_ by quantifying the correlation between activities evoked by two simultaneously presented movie patches ( Figure 5B, see arrows). All correlation values were higher than corresponding r _single_ values ( Figure 5, *red* dots). r _coherent_ was equal to 0.84 [0.74, 0.89], resulting in an increase of 46.9%. This result suggests that long-range interactions increase the similarity of the activity time-course. In accord with this conclusion, we observed that an incoherent movie-patch presented simultaneously had a detrimental effect on the correlation values. Consequently, r _incoherent_ was 30% smaller than r _coherent_ and equal to 0.58, ([0.38, 0.74], Figure 5B, *black* dots). This result suggests that long-range interactions, in addition to their facilitatory effects, lead to an increase in the similarity of the time-course.

Experiment Raw data of all the 4 experiments presented in this report are made available in Matlab format. These files contain a matlab structure named EVO that represents the evoked activity and related information. The field Evo.d contains the data and is organized as [space, time, condition]. The total length of the space dimension equals to Evo.ys*Evo.xs; the third dimension contains different conditions and is organized as follows: 1-2: Blank Conditions, 3: Condition A1B1, 4: A1, 5: B1, 6: A2B2, 7: A2, 8: B2, 9: A1B2, 10: B2A1. In order to plot the Nth sample of the Mth condition, one would need to issue the following command in Matlab: "imagesc(reshape(evo.d(:,N,M),evo.ys,evo.xs))4

Video S1: Natural movies and stimulus conditions. Two different natural movies and derived local stimulation conditions are shown as they were used during one of the experiments. To facilitate inspection, movies are shown at half of the speed (12 Hz) used during the experiments. The right-hand color labels (red/blue) code for condition identity. The monitor covered a visual field of approximately 30×40 degrees.

Video S2: Activation patterns during locally presented natural images. Evoked optical activity during local stimulation with a single or a pair of natural movie patches. First and third rows show full-field natural movies (left) and derived local conditions. Second and fourth rows depict cortical activity patterns measured with VSDI. The scale bar represents 1 mm.

## Discussion

We used VSDI to investigate long-range cortical interactions during processing of natural images in the primary visual cortex at the mesoscopic population level. By using "keyhole-like" presentations of the original natural movies through either one or two distant Gaussian masks, we quantified the effect of surrounding stimulation on local activity. We provide evidence that contextual integrative mechanisms are indeed operative under natural stimulus conditions. We show that under these conditions the horizontal cortical network ^34– 37^ forms the basis for synergistic interactions across several millimeters of cortex. Contextual stimulation led to a net facilitatory effect compared to the case when the movies were shown in isolation. An important attribute of these interactions was their sensitivity to the intrinsic spatiotemporal regularities of natural movies ^38, 39^. Moreover these contextual interactions led to an increased similarity of the population dynamics across long-range cortical distances.

Contextual processing has been investigated extensively both experimentally at the single neuronal level ^40^ and in recent modeling approaches ^41^. A large variety of facilitatory and/or inhibitory contextual effects have been observed, however the final outcome crucially depends on the precise configuration of the parametrized stimulus used to stimulate center and surround regions. While the surround effect was found to be mainly inhibitory ^35, 42, 43^ and spatially asymmetrically organized ^44^, the precise nature of the effect depends on the contrast of contextual stimuli relative to the contrast threshold of the recorded neuron ^45– 47^.

An important cornerstone of long-range facilitation is its dependence on the precise spatial configuration of the surrounding context ^48^. It has been shown that facilitatory effects increase proportionally with the congruency of the contextual stimuli with respect to the center stimulus ^18, 49^. Using static stimuli, such coherence is generally controlled parametrically by changing the orientation difference between center and surround patches ^17, 18, 50, 51^. Since we here used natural movies recorded by cats that freely explored a natural habitat, our stimuli were complex and contained simultaneous multiple features. The head and body movements of the cats added to this complexity as the recorded visual stimuli contained motion cues that were correlated across large visual distances. In order to control the coherency of the stimuli between the apertures, we adopted a non-parametric method by exploiting the unique spatiotemporal characteristics of each original movie.

With surround gratings-dependent on their relative orientation, distance, and contrast-both facilitatory ^17, 18, 42, 45, 48, 49, 52, 53^ and suppressive neuronal effects ^17, 18, 42, 45, 50, 52, 54– 57^ on a center stimulus have been described in the literature. In contrast, using natural stimuli in our study, we exclusively observed facilitation. Interestingly in this regard, when using randomly placed oriented lines as contextual stimuli, Kapadia and colleagues (1995) found that “inhibition could be eliminated by changing the orientation of a few of these elements”. This led them to suggest that with the “appropriate configuration of contours surrounding the RF, the cell is lifted from a rather profound level of inhibition, and its excitatory inputs are unmasked”. In this sense, the specific regularities in our naturalistic movies could be viewed as “appropriate configuration” and were shown here to trigger facilitatory interactions across long-range cortical distances. Whether these super-additive interactions result from disinhibition ^49^ or from additional excitatory drive through “cross-orientation” mechanisms remains to be explored.

When the same movie was presented through both apertures they were perceptually grouped without effort, and appeared to belong to a single scenery. On the other hand, when two differing movies were used, the content within both apertures appeared to be immediately incompatible (please see Video S1). There are a number of factors that determine coherence between patches taken from the same movie. First, stimulus motion was similar between the two distant apertures. This was due to the body- and head-motions of the recording cat, which induced large and equal motion fields across the visual scene captured by the camera. It has been earlier noted that such temporal phase relationships across distant regions are perceptually salient and enable object segmentation even in the absence of any spatial information ^58^. Second, natural images tend to possess large spatial correlations because of the dominance of low spatial frequencies in their spectrum ^21^. Moreover auto-correlations of orientations may cover large portions of visual field reaching up to 8 degrees ^59^. Therefore, our stimulus paradigm can be conceived as a dynamic illustration of Gestalt criteria of good continuation.

There are different idealized mechanisms, each based on different anatomical substrates, which could mediate the observed facilitatory long-range interactions. Overlapping feed-forward thalamo-cortical input could be an explanation for increased cortical drive during stimulation with adjacent movie-patches. However, there are a number of counter-arguments against this explanation. First, cortical locations driven most strongly by the individual movies were separated by distances larger than the anatomical spread of direct thalamo-cortical projections ^5, 15, 60, 61^. This was in accord with relatively smaller spatial extents of mapped receptive fields. Second, the activity at the distant location during stimulation with one single movie-patch was only minimal and reached significant levels about 50 ms later in comparison to directly stimulated locations. Third, and most decisively, the total drive to the recorded cortical area was constant across the two coherent and incoherent conditions. Only the order of the presentation being different, it is not possible to account for facilitatory interactions in a purely feed-forward scheme.

Rather, the dense network of horizontal connections linking distant neurons across several millimeters is a likely candidate for the observed long-range effects. It has been shown that unmyelinated intralaminar connections contribute to subthreshold responses evoked from distant stimuli placed outside of the classical receptive fields both with intracellular ^5^ and combined extracellular recordings and VSDI in cat ^7^. Furthermore, the selective intracortical connectivity pattern of these tangential connections linking neurons with similar feature selectivity is well-suited to mediate the specific enhancement of activity levels dependent on the stimulus coherence. However, we cannot exclude that feedback signals originating from higher visual areas with larger receptive field sizes than in primary visual cortex could add to these interactions ^62, 63^. Back-propagating waves of activity have been shown to be initiated in further downstream cortical areas as early as ~100 ms after stimulus onset ^64, 65^. Thus, these connections act fast ^66, 67^ and are likely to mediate surround modulations spanning considerable distances in visual space, while lateral intra-laminar connectivity may account for modulations within shorter distances ^68^.

The relationship between the VSD imaging signal and spiking activity can indeed be complex. For instance, a close relationship between spike rate and the derivative of the VSD response rather than its magnitude has been proposed ^69^. This might especially apply to the rising phase of the membrane potential after stimulus onset ^6, 70^. Combined VSD and Calcium-sensitive dye imaging suggests that the relationship between spiking activity and the amplitude of the VSD response depends also on stimulus intensity ^71^. However, Chen *et al.* ^72^ found that these relationships are well-captured by a power function with an exponent of ~4 (similarly as for the relationship between average membrane potential and spike rates in single V1 neurons ^73, 74^), indicating that the threshold for observing significant spiking activity is about 30–40% of the maximal VSD response. Amplitudes evoked by our natural movies were on average well-within this range as compared to full-field moving gratings that we used as a standard control for maximal stimulation. Moreover, only pixels fitted at the center of the movie representations with highest amplitude entered our analysis, furthermore ensuring that subthreshold activity was excluded. Thus, we are confident that we describe the effect of long-range connectivity on postsynaptic spiking activity.

We observed that, compared to incoherent stimulation, stimulation with coherent pairs of natural stimulus patches led to stronger facilitatory effects. Since the total input analyzed across coherent and incoherent stimulation was identical across the recorded cortical area, these results cannot be solely explained by the local properties of movie-patches. Rather, this facilitation necessarily reflects the outcome of an integrative phenomenon sensitive to the content of both local movie-patches when presented simultaneously. Therefore, we suggest that the functional architecture of early visual cortical circuits may have empirically internalized the typical contextual relationships ^21– 23^ found in dynamic natural visual scenes.
