# Supplementary material for: Cortical long-range interactions embed statistical knowledge of natural sensory input: a voltage-sensitive dye imaging study
Source: F1000Res. 2013 Aug 2;2:51. Originally published 2013 Feb 15. [Version 2] doi: 10.12688/f1000research.2-51.v2 (PMC3829195; doi:10.12688/f1000research.2-51.v2)
Supplement: Peer review history

# Peer review: Referee response for version 2

**DOI:** 10.5256/f1000research.1959.r3974

**Published:** 2014-03-04

**Authors:** Diego Contreras

**Affiliations:** 1 Department of Neuroscience, University of Pennsylvania School of Medicine, Philadelphia, PA, USA

I have read this submission. I believe that I have an appropriate level of expertise to confirm that it is of an acceptable scientific standard.

# Peer review: Referee response for version 2

**DOI:** 10.5256/f1000research.1959.r3280

**Published:** 2014-01-23

**Authors:** Marcello Rosa

**Affiliations:** 1 Faculty of Medicine, Monash University, Melbourne, Victoria, Australia

I have read this submission. I believe that I have an appropriate level of expertise to confirm that it is of an acceptable scientific standard.

# Peer review: Referee response for version 1

**DOI:** 10.5256/f1000research.1179.r811

**Published:** 2013-03-06

**Authors:** Diego Contreras

**Affiliations:** 1 Department of Neuroscience, University of Pennsylvania School of Medicine, Philadelphia, PA, USA

1. What is the point of using “ecological” stimuli if the response is then reduced to 6 parameters? How is that different from using drifting gratings? The authors should either provide the comparison with simpler stimuli or better justify their choices. The main point here is that the larger summation of two stimuli from the same movie versus two stimuli from different movies may be simply due to objects that span the two masks generating a similarity in the orientation and spatial frequency in the two stimuli, which, in turn, would lead to very well-known orientation-specific effects of contextual stimuli. 
2. How is presenting another stimulus “contextual”? the stimuli where either one or two stimuli of identical size. It would be easier to read if the authors used only one terminology, such as single, coherent and incoherent. 
3. How was a 2D Gaussian fit to the response of one of the stimulus while presenting both? The response is merged, doesn't this affect the fitting procedure?
4. What was learned from the multi-unit recordings?
5. Figure 2: It is pretty but mostly useless without quantification, the authors should show values of dF/F or contour plots of the significant response (3 or 5 SDs above baseline noise) so that the movies can be properly compared. 
6. It is remarkable that the responses to the same mask in movie 1 and 2 are so different, are they really statistically different? Is the VSD signal capable of discriminating the differences between the two movies? Looking at the movies it seems that they have similar spectrotemporal composition.
7. Where were the values presented on top of page 5 taken from? What area? What time?
8. Figure 4:What is “modelized”? Does figure 4A not represent data?
**Minor Points:**
9. Page 4, 2nd column: Dissonance? 
10. What is “superadditive”? Does it mean supralinear? Very difficult to understand, mainly because the term is used in combinations such as “superadditive facilitation”

I have read this submission. I believe that I have an appropriate level of expertise to confirm that it is of an acceptable scientific standard.

# Response

**Published:** 2013-08-09

**Authors:** Selim Onat

**Affiliations:** University Medical Center Hamburg-Eppendorf, Germany

Point by point response to Dr Contreras: 

1. These are important questions (please also see our response to question 3 from Dr Rosa below). Clearly, there exist a remarkable number of studies (references in main text) about contextual effects using local grating patches of certain orientations and spatial frequencies.
  In our work we follow instead the hypothesis that the low-level sensory regions (if not the whole nervous system) are wired to optimally process inputs that are most likely to be received from the sensory organs. Our recent report ^26 ^suggests that the cortical state (i.e. both the dynamic range and mean activity) is qualitatively different when stimulated with gratings as compared to ecological stimuli. Thus, response behavior to natural input can deviate significantly from predictions based on simple parameterized stimuli, probably due to the extensive spatial information in natural images ^22, 29^_._
  These observations may place the burden of justification on the use of simple stimuli, and not of natural stimuli as used in the present report. Many intra-areal long-range facilitation phenomena in the early visual system have been shown to occur in response to local presentations of simple stimuli. The evidence showing that these are also functional under more natural stimulation conditions that are more complex and also more ecological is not clear. For instance, both the extent and the impact of long-range connections may critically depend on the correlation structure across neuronal populations ( Lindén *et al*., 2011), which in turn emerges from the specific spatio-temporal correlations of visual input. Therefore, in this manuscript, we aimed to demonstrate that long-range interactions are also functional and effective under more naturalistic stimulation conditions, which were so far not investigated. We provide this evidence in this manuscript and we have added a paragraph to the discussion section (paragraph 4) of our manuscript.
  Finally, the choice of a low number of parameters to characterize the response is data driven. We demonstrate that our analysis captures the larger part of variance of the response as obtained by voltage sensitive dye imaging (VSDI) and is therefore a valid procedure. A comparison of the dimensionality of stimulus and response space has to take into account the non-linearity of processing in the visual system. 
2. Our use of “contextual” is inspired by usage of the terms classical receptive field and non-classical receptive field/context in the visual sciences: Here one visual stimulus elicits a reliable response at a certain location. The other stimulus does not elicit detectable activity at that location, but as our data demonstrate modulates the response to the former stimulus. The term “context” is intended to denote that dependence. That is, we consider this neighboring information as a contextual input as it doesn’t directly influence the processing at the distant center. 
3. In all cases, including single stimulation conditions, there were two 2D Gaussians that were combined to fit the observed responses. The parameters were estimated simultaneously. Using two Gaussians in all stimulation conditions ensured that the activation at the distant locations did not influence the fit. Most of the time, the activity spots were clearly distant and well-isolated from each other, therefore possible cross-contributions were negligible. Furthermore the center position of each single Gaussian fit was constrained around the location of the peak responses at locations A and B. This helped avoiding complications where Gaussians could overfit noise. We now included this information in the manuscript (Methods, page 8, last paragraph). 
4. Electrical recordings were performed to allow for rapid retinotopic hand mapping of the imaged area before VSDI. Electrical recordings parallel to VSDI were done to verify occurrence of evoked spiking activity at those cortical locations where high levels of the dye signal was observed, i.e. around the center representation of one of the local movie patches. Note that a single recording site was used. Thus, analysis of reciprocal interactions at two locations simultaneously, as resolved by VSDI, was not feasible. Analysis of the electrical recordings related to particular stimulus features will be subject of a following report. 
5. The colorbar depicts x10 ^-4^ dF/F values. This is now added to the legends of Figures 2 to 4. Pixels with an activity smaller than 1.5x10 ^-4^ dF/F are not shown. This corresponds to the 75 ^th^ percentile of the activity distribution, as noted on the figure legend. Responses of each single movie are depicted with their own colormap so that the precise detailed structure of the evoked responses can be clearly seen for each single condition. Using a common color scale would basically eliminate the possibility to clearly see responses to movies that evoked weaker responses. Furthermore, the colorbar helps comparison of amplitudes across different movies and positions. 
6. VSDI can clearly distinguish these differences. In Onat *et al.* ^13^, we have shown that VSDI can distinguish diverse stimulus related components in response to drifting gratings including actual position, i.e. the retinotopic trajectory, of the grating stripes. In the current manuscript, in Figure 2, it is also possible to see that the same movie during double or single conditions leads to similar temporal activity profiles (as analyzed in Figure 5). 
7. These are peak values computed across the entire stimulation duration and all recorded pixels. 
8. Figure 4A (top row) presents the spatial activity profiles observed on the cortical surface that are reconstructed using the Gaussian parameters (mu, sigma, amp) averaged across ROIs and cats. Please notice that averaging the raw recorded data across cats and ROIs (A and B) is not possible. This is due to the large differences in the shape of the activity profiles across different ROIs and cats, therefore we reconstructed these activity profiles using the fitted parameters. Each individual data point that contributes to these spatial profiles are depicted in Figure 4B. We replaced the word “modelized” with “modeled”. You might view this analysis technique as a low dimensional parametric description of the experimental data. 
**Minor Points**
9. We used the term dissonance to describe the perceptual quality of movie patches originating from different movies when shown simultaneously. 
10. We used the term super-additive, to indicate a response that is more than the sum of the two individual components when these individual components are presented simultaneously. The meaning is essentially the same as supralinear, although in the literature super-additive is used more often. 

*Reference numbers refer to the reference list in the manuscript*

# Peer review: Referee response for version 1

**DOI:** 10.5256/f1000research.1179.r784

**Published:** 2013-02-19

**Authors:** Marcello Rosa, Nicholas S Price, Hsin-Hao Yu

**Affiliations:** 1 Faculty of Medicine, Monash University, Melbourne, Victoria, Australia; 2 Department of Physiology, Monash University, Melbourne, Australia

1. The authors seem to only deal with linear addition of activation. We are not sure that this is a reasonable assumption, without more information about how optical activity scales with spiking rate. Thus, supra-linear summation may not be that surprising. i.e. the presence of one stimulus patch might lift the optical activity to close to threshold, so a second patch then seems to create a lot of activation, but it's just because there was a "hidden" threshold. 
2. Figure 5 is pretty complicated. We had a hard time understanding it. Maybe it needs to be broken into smaller, more digestible bits of information and have a clearer legend.
3. This paper will be more useful if they also present data using gratings as stimuli.

We have read this submission. We believe that we have an appropriate level of expertise to confirm that it is of an acceptable scientific standard.

# Response

**Published:** 2013-08-02

**Authors:** Selim Onat

**Affiliations:** University Medical Center Hamburg-Eppendorf, Germany

Point by point response to Dr  Rosa: 

1. This is an important point. The relationship between the VSD imaging signal and spiking activity can indeed be complex. For instance, a close relationship between spike rate and the derivative of the VSD response rather than its magnitude has been proposed ^69^_._ This might especially apply to the rising phase of the membrane potential after stimulus onset ^6,70^. Combined VSD and Calcium-sensitive dye imaging suggests that the relationship between spiking activity and the amplitude of the VSD response depends also on stimulus intensity ^71^. However, Chen *et al.*, ^72^ found that these relationships are well-captured by a power function with an exponent of ~4 [similarly as for the relationship between average membrane potential and spike rates in single V1 neurons ^73,74^, indicating that the threshold for observing significant spiking activity is about 30–40% of the maximal VSD response. Amplitudes evoked by our natural movies were on average well-within this range as compared to full-field moving gratings that we used as a standard control for maximal stimulation. Moreover, we compared the fitted peak amplitudes and this ensures that only pixels with highest amplitude at the center of the movie representations entered our analysis. Therefore, we are confident that subthreshold activity was excluded and that we describe the effect of long-range connectivity on postsynaptic spiking activity. We added this information to Discussion (page 10, before the last paragraph) 
2. Dividing the Figure 5 might make it more difficult to understand given that the data that is shown on the scatter plots are summarized on the top panels, which represents the average observations. 
3. Interestingly, using LFP as a population measure of postsynaptic activity, a recent study showed that superimposed gratings (i.e. plaids) lead to responses that were not predicted by activity in response to their single components ( Bartolo *et al*., 2011). Thus, a simple parametric manipulation (addition) of most simple stimuli like gratings can lead to drastic changes in neuronal population response behavior limiting explanatory power. If this is the case, the use of gratings may be a weak predictor with respect to complex stimulation, in particular with respect to natural scenes. Please also see above for our reply to Dr Contreras who had a similar concern.

*Reference numbers refer to the reference list in the manuscript*
